# Use of virus-like particles and nanoparticle-based vaccines for combating picornavirus infections

**DOI:** 10.1186/s13567-024-01383-x

**Published:** 2024-09-30

**Authors:** Mei Ren, Sahibzada Waheed Abdullah, Chenchen Pei, Huichen Guo, Shiqi Sun

**Affiliations:** 1 State Key Laboratory for Animal Disease Control and Prevention, CollegeofVeterinaryMedicine, Lanzhou UniversityLanzhou Veterinary Research InstituteChinese Academy of Agricultural Sciences, Lanzhou, 730000 China; 2 Gembloux Agro-Biotech, University of Liege, Gembloux, Belgium; 3 Livestock and dairy development department peshawar, Government of Khyber Pakhtunkhwa, Peshawar, Pakistan

**Keywords:** Virus-like particles, nanoparticles, adjuvant, vaccine, picornavirus

## Abstract

*Picornaviridae* are non-enveloped ssRNA viruses that cause diseases such as poliomyelitis, hand-foot-and-mouth disease (HFMD), hepatitis A, encephalitis, myocarditis, and foot-and-mouth disease (FMD). Virus-like particles (VLPs) vaccines mainly comprise particles formed through the self-assembly of viral capsid proteins (for enveloped viruses, envelope proteins are also an option). They do not contain the viral genome. On the other hand, the nanoparticles vaccine (NPs) is mainly composed of self-assembling biological proteins or nanomaterials, with viral antigens displayed on the surface. The presentation of viral antigens on these particles in a repetitive array can elicit a strong immune response in animals. VLPs and NPs can be powerful platforms for multivalent antigen presentation. This review summarises the development of virus-like particle vaccines (VLPs) and nanoparticle vaccines (NPs) against picornaviruses. By detailing the progress made in the fight against various picornaviruses such as poliovirus (PV), foot-and-mouth disease virus (FMDV), enterovirus (EV), *Senecavirus* A (SVA), and encephalomyocarditis virus (EMCV), we in turn highlight the significant strides made in vaccine technology. These advancements include diverse construction methods, expression systems, elicited immune responses, and the use of various adjuvants. We see promising prospects for the continued development and optimisation of VLPs and NPs vaccines. Future research should focus on enhancing these vaccines' immunogenicity, stability, and delivery methods. Moreover, expanding our understanding of the interplay between these vaccines and the immune system will be crucial. We hope these insights will inspire and guide fellow researchers in the ongoing quest to combat picornavirus infections more effectively.

## Introduction

According to the International Committee on Taxonomy of Viruses (ICTV), *Picornaviridae*, a large family of non-enveloped, single plus-strand RNA viruses, comprises 68 genera and 158 species. It can infect humans and many other vertebrates (as of March 2024) [1–3]. Members of this family have thus far caused great harm to human society. Such perpetrators include the famous poliovirus (PV), which causes poliomyelitis, enterovirus 71 (EV71) and Coxsackie A virus 16 (CVA16), which both cause hand-foot-and-mouth disease, a childhood infectious disease often occurring in children under 5 years old. Other examples include hepatitis A virus (HAV), which causes hepatitis A, encephalomyocarditis virus (EMCV), which causes infectious diseases such as encephalitis, myocarditis, or diabetes; and foot-and-mouth disease virus (FMDV), which causes foot-and-mouth disease (mainly infecting cloven-hoofed animals such as cattle and pigs) [4–9]. When the host is exposed to these viruses, the immune system initiates a series of complex immune responses to clear the infection and produce long-lasting immune memory. Once picornaviruses enter the host, they can replicate and cause diseases in the respiratory tract, intestines, or other tissues [10]. Next, the animal’s immune system quickly responds to this viral invasion. This immune response includes two main types: innate immunity and adaptive immunity. Innate immunity is the body’s primary barrier and includes the skin, mucosa, inflammatory response, and various immune cells, such as natural killer cells (NK cells) and neutrophils [11]. These innate immune mechanisms attempt to clear the virus or prevent its further spread directly. For example, the mucus on the mucosal surface can attach to and prevent viruses from entering cells, while neutrophils can engulf and eliminate infected cells. Adaptive immunity is a more complex and specific immune response, predominantly involving thymus lymphocytes (T-cells) and bursa-dependent lymphocytes (B-cells). When picornaviruses infect cells, the viral proteins are released and presented to specific T-cells for recognition. After identifying infected cells, T-cells can kill these cells, thereby limiting the spread of the virus. At the same time, infected cells release signalling molecules, attracting other cells in the immune system to help, such as macrophages and NK cells that engulf viruses. During this process, B-cells are activated and produce antibodies that can specifically bind and neutralise picornaviruses, preventing them from continuing to infect other cells [12]. Overall, infection with picornaviruses can trigger complex immune responses, including innate and adaptive immune responses.

These immune responses aim to clear infections, limit virus spread and generate long-lasting immune memory that will protect the host from future infections. However, more persistent infections can still occur within the picornavirus family, such as with FMDV. Currently, persistent infection of FMDV mainly occurs in animals, in which the virus remains present without clinical symptoms despite recovery from acute FMD infection [13, 14]. Ruminants often tend to develop persistent infections after initial infection with FMDV. Cattle can experience persistent infections that last up to three and a half years [14, 15]. Contact between these persistently infected animals and susceptible animals can inevitably lead to a disease outbreak. Related studies have indicated that injecting saliva from animals with persistent FMDV into healthy cattle and pigs results in infection [15]. Fortunately, it is also reported that the likelihood of persistent infection with the FMD virus decreases if the immune response to vaccination has developed adequately [16].

Vaccines as biological products mainly aim to activate the immune system by prompting specific immune responses, in turn enhancing resistance to particular pathogens and preventing, or reducing, the incidence, severity, and mortality of corresponding diseases [17]. The immune system can recognise and generate antibodies or cellular immune responses against pathogens without actual infection through vaccination and immune memory combined. This enables the body to quickly and effectively respond to infections and protect itself from diseases when encountering the pathogen. During the COVID-19 pandemic, vaccination remained the most effective defence method [18]. The success of the lipid nanoparticle COVID-19 mRNA vaccine blazed a trail for the application of nanotechnology in vaccine development.

Research on picornavirus vaccines is considered one of the most important areas of study. In recent years, new-generation vaccines have emerged quickly because the live virus does not need to be used in production, which is a big advantage [19]. During this century, alongside the development of new vaccines and nanotechnology, research on virus-like particles (VLPs) and nanoparticles (NPs) vaccines has increased exponentially [20]. VLPs refer to spherical or tubular protein nanostructures formed by the self-assembly of viral structural proteins. Compared to intact viruses, VLPs lack a viral genome and are not infectious [21]. NPs largely comprise self-assembling biological proteins or nanomaterials, with virus epitopes repeatedly displayed on the surface [22]. These particles have thus far attracted much attention as they hold several advantages. They can achieve slow and sustained antigen release and also serve as adjuvants because they can, at the same time, act as antigen delivery systems and innate immune response stimulants [23–26]. VLPs and NPs have been widely used in research on virus vaccines [27–29]. Many studies have recently been published that focus on developing VLPs and NPs vaccines against picornaviruses (Tables 1 and 2) [29–34].

**Table 1:** **The VLPs vaccine of Picornavirus**

Virus	Composition	Expression system	Year of publication	Reference
PV	cDNA	B/IC: *Sf*-9	1989	[84]
	P1-3CD	Hela	1991	[83]
	P1-3CD	*Saccharomyces cerevisiae*	1997	[85]
	P1-3CD	*Nicotiana benthamiana*	2017	[86]
	P1-3CD	*Pichia pastoris*	2020	[87]
	P1-3CD	BHK-21	2021	[89]
	P1, P2A peptide	*Pichia pastoris*	2023	[90]
FMDV	cDNA	B/IC: *Sf*-9	1990	[91]
	P1-2A, 3C	B/IC: *Sf*-9	2009	[94]
	VP0/3/1	*Escherichia coli*	2009	[97]
	P1-2A, 3C	B/IC: *Sf*-9	2013	[95]
	P1-2A, 3C	B/IC: *Sf*-9	2013	[96]
	VP0/3/1	*Escherichia coli*	2013	[29]
	P1-2A, 3C	B/IC: *Sf*-21	2015	[98]
	P1-2A, 3C	*Samia cynthia ricini*	2016	[99]
	P1-2A, 3C	*Nicotiana benthamiana*	2018	[100]
	P1-2A	*Agrobacterium tumefaciens*	2018	[101]
	VP0/3/1	*Escherichia coli*	2019	[106]
	P1-2A, 3C	HEK-293A	2020	[102]
	P1-2A, 3C	HEK-2936E	2020	[103]
	VP0/3/1	*Escherichia coli*	2022	[108]
	P1-2A, 3C	HEK-293 T	2022	[104]
	P1-2A, 3C	BHK-21	2023	[105]
EV71	P1-3CD	B/IC: *Sf*-9	2008	[80]
	P1-3CD	*Saccharomyces cerevisiae*	2013	[112]
	P1-3CD	HEK-293A	2015	[113]
	P1, 3C	*Flammulina velutipes*	2015	[114]
	P1, 3C	*Pichia pastoris*	2020	[110]
	P1, 3CD	*Pichia pastoris*	2023	[192]
SVA	VP0/3/1	*Escherichia coli*	2020	[115]
	VP1-VP3, VP2-VP4	B/IC: *Sf*-9	2024	[117]
EMCV	P1-2A, 3C	HEK-293FT	2010	[118]
	VP0/VP1/VP3	*Escherichia coli*	2024	[193]
CVA16	P1-3CD	B/IC: *Sf*-9	2012	[120]
CVB3	VP1/VP2/VP3/VP4	B/IC: *Sf*-9	2012	[121]
DHAV	P1, 3CD	B/IC: *Sf*-9	2018	[119]

**Table 2:** **The NPs vaccine of picornavirus**

Virus	Antigen	Particle skeleton	Formation	Year of publication	Reference
FMDV	B_4_T (B: VP1(Residues 136 aa-154 aa) T: 3A (Residues 21 aa-35 aa))	B_4_T	Synthesised	2008	[130]
	P1, 3C	T4 bacteriophage	*Escherichia coli*	2008	[155]
	B_4_T, B_2_T	B_4_T, B_2_T	Synthesised	2016	[133]
	G-H loop	T7 bacteriophage	*Escherichia coli*	2017	[160]
	B_4_T, B_2_T (B: VP1(Residues 135 aa-160 aa) T: VP1 (Residues 21 aa-40 aa))	B_4_T, B_2_T	Synthesised	2017	[134]
	VP1, G-H loop	Ferritin	B/IC: *Sf*-9	2020	[34]
	Residues 16 aa-44 aa, 129 aa-160 aa and 200 aa-213 aa (VP1)	ADDomer	B/IC: *Sf*-9	2022	[165]
EV71	Residues 208 aa-222 aa, 215 aa-218 aa, 213 aa-220 aa in VP1	Ferritin	*Escherichia coli*	2019	[150]

This review describes the immune responses related to picornaviruses. It explains why VLPs and NPs research is important for developing vaccines against this virus family, and describes the development history of VLPs and NPs research to help combat picornaviruses such as PV, FMDV, EV, EMCV, and Senecavirus A (SVA). It explores their construction, expression, immune responses elicited, and the adjuvants used. In brief, VLPs and NPs vaccines are up-and-coming vaccine development platforms that display good safety, immunogenicity, stability, and the ability to display exogenous proteins. Future research should now focus more on their design scheme, targeting, biological distribution, and potential cytotoxicity. Artificial intelligence can be applied to design scaffold structures or optimal epitope presentation strategies.

## Immune responses against picornaviruses

### Innate immunity

**Figure 1:**
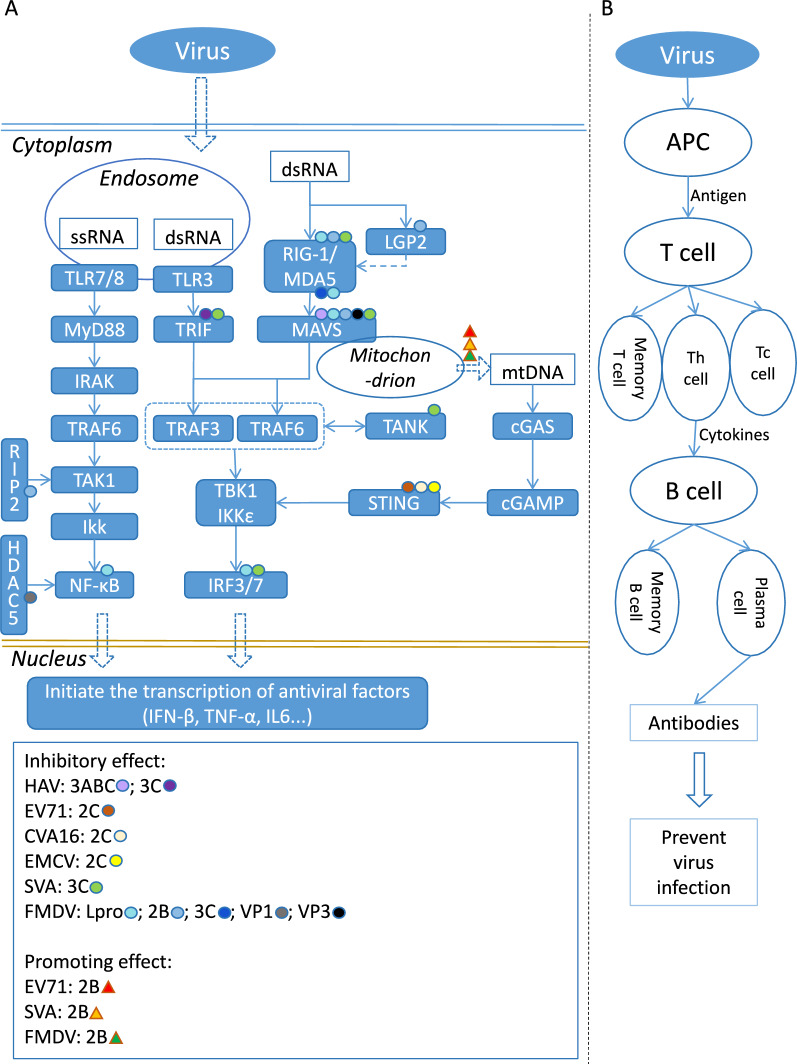
**Immune responses against picornaviruses.** **A** Innate immunity induced by picornavirus infections, which includes Toll-like Receptors (TLRs) signalling pathway, retinoic acid-inducible gene I (RIG-1) signalling pathway, and cyclic GMP-AMP synthase (cGAS) signalling pathway. Also, the innate immune escape mechanisms of picornaviruses were indicated; **B** Acquired immunity induced by picornavirus infections. After the virus enters the body, it is captured and processed by antigen-presenting cells (APCs), which present the processed antigen to T-cells. T-cells are activated. Subsequently, activated T-cells secrete cytokines and activate B-cells. B-cells differentiate into plasma cells and produce many specific antibodies. Cytotoxic T-cells (Tc cells) directly kill infected cells. Helper T-cells (Th cells) enhance the overall immune response by secreting cytokines. Another part of the T-cells and B-cells differentiate into memory cells and remain in the body for a long time, so that when the body is exposed to the same pathogen again, the memory cells respond quickly and provide a faster and stronger immune response.

Both innate immunity and acquired immunity are involved in picornavirus infection. Innate immunity is the host’s first defence mechanism against pathogen infection. It recognises invading pathogens through pattern recognition receptors (PRRs) and activates signal pathways such as interferons (IFNs), NF-κB, and inflammasomes. These innate immune signalling pathways do not exist independently but instead interact. In picornaviruses, the innate immune system recognises viral double-stranded RNA (dsRNA) through two distinct pathways: the Toll-like receptors (TLRs) pathway, which detects dsRNA phagocytosed in endosomes and the helicase retinoic acid-inducible gene I protein (RIG-1), which detects cytoplasmic dsRNA generated during viral replication [35]. As these receptors sense most picornaviruses, the interactions between the proteins of these pathways and the viral proteins have been investigated in detail. Members of this virus family can often evade the innate immune responses by degrading these receptors or downstream molecules in the signalling pathways (Figure 1A). It has been reported that both structure and non-structure proteins of picornaviruses can inhibit the TLRs signalling pathway and the RIG-1 signalling pathway [36–44]. For example, HAV’s 3ABC protein, a precursor of 3C protease, targets MAVS for cleavage and thus disrupts the RIG-I/MDA5 signalling pathway [45]. 3CD, another of HAV’s precursors of 3C protease, can disrupt the TLR3 signalling pathway by degrading TRIF [46]. For FMDV, it has evolved multiple strategies to evade the immune response, particularly the type I IFN response [47]. The viral nonstructural protein Lpro cleaves NF-κB and IRF3, which are both crucial for inducing IFN-β mRNA [48]. The 3Cpro inhibits MDA5 protein expression through its protease activity, thus inhibiting the production of type I interferon. FMDV structural protein VP1 degrades histone deacetylase 5 (HDAC5) via the proteasomal pathway, while HDAC5 acts as a positive modulator of IFN-β production during viral infection [49]. For SVA, its nonstructural protein 3C plays a role in this process. The 3C protein can cleave and degrade critical components of the PRR signalling pathways, including RIG-I, MAVS, IRF3, IRF7, TRIF, and TANK, thereby suppressing the production of IFN [50]. Additionally, it has been reported that cyclic GMP-AMP synthase (cGAS) plays a key role in the innate immune responses to picornavirus infection. During EV71, SVA, and FMDV infection, their 2B proteins trigger the release of mitochondrial DNA (mtDNA) [51]. The released mtDNA binds to cGAS and activates a cGAS-mediated antiviral immune response. At the same time, the 2C proteins of EV71, CVA16, and EMCV antagonise the cGAS-stimulator of interferon genes (STING) pathway by interacting with STING. In conclusion, picornaviruses use their own structural and nonstructural proteins to antagonise, inhibit, or degrade various host proteins in the innate immune signalling pathway during infection. They interfere with the posttranslational modification of host proteins in the innate immune process and block the host's innate immunity in various ways, resulting in low host immunity, enabling the virus to replicate rapidly and continue to infect.

### Acquired immunity

Acquired immunity is crucial in defending against pathogens by generating specific responses tailored to the encountered threat. Acquired immunity is also called “specific” or “adaptive” immunity. It describes the host’s ability to resist infection through acquired infection or vaccination. It is generally achieved after infection or the stimulation of other antigenic substances, where the host reacts specifically with this antigen (Figure 1B). The adaptive immune system is mainly composed of two types of lymphocytes: B-cells and T-cells [52]. B-cells can differentiate into plasma cells after recognising specific antigens, and the plasma cells produce and release many antibodies. These antibodies can bind to pathogens, a key process for fighting the picornavirus infection. Another part of the B-cell differentiates into memory B-cells, which quickly react when exposed to the same antigen again. Helper T-cells (Th cells) aid the activation and function of other immune cells, including B-cells and cytotoxic T-cells. Th cells enhance immune responses by releasing cytokines. Specifically, we must emphasise the critical role of T-cells in facilitating efficient B-cell responses, including by generating memory B-cells. An adequate level of T-cell assistance is essential for the activation, proliferation, and differentiation of B-cells, which are crucial for robust and long-lasting antibody production. These interactions between B-cells and T-cells become even more critical in the design of NPs, where a reduced number of B- and T-cell epitopes are frequently included [53]. The T-cell epitope is insufficient or suboptimal, potentially impacting the efficiency of B-cell activation and subsequent immune memory formation. Therefore, T-cell-mediated activation signals are needed for B-cell maturation, and memory B-cell development is crucial for the design of NPs. Furthermore, the quantity and density of antigens displayed on NPs are closely related to the vaccine’s effectiveness. Existing data shows that displaying more and denser antigens can activate more B-cells, but at the same time, an excessive density can affect the effectiveness of antigen binding [54, 55]. Therefore, the balance between quantity and distance must be maintained. By elucidating these mechanisms, we aim to provide a more explicit rationale for the design of NPs and underscore the importance of optimising epitope selection to ensure robust adaptive immunity.

For protection against picornavirus infection, the binding of neutralising antibodies (nAbs) to the icosahedral capsid is essential, as it prevents virus entry into cells and, in turn, prevents infection [22, 56]. This protection mainly comes from the plasma cells produced by the antibody binding to the epitope, which inhibits viral attachment and entry into cells. After binding, viral attachment can be inhibited through steric hindrance or changes in capsid conformation that abrogate interactions. These conformational changes in the capsid can also stop infection by changing the capsid’s stability, leading to the early extracellular release or intracellular non-release of viral RNA.

Therefore, designing a vaccine that can stimulate a large amount of nAbs is one of the most effective methods of preventing picornavirus infection. However, as an RNA virus, the replication of picornavirus is primarily mediated by its RNA-dependent RNA polymerase (RdRP), which lacks an efficient proofreading mechanism [57, 58]. This leads to a high genetic and antigenic variability amongst picornaviruses. This characteristic further complicates the design of vaccines for picornaviruses [59]. Nevertheless, VLPs and NP vaccines, due to their short production cycles and relatively simple production processes, can be rapidly and efficiently designed and produced to target newly emerging epidemic strains. As a result, they have become a focal point in picornavirus vaccine research [60].

With the emergence of nanotechnology, VLPs and NPs have become popular choices for designing vaccines against picornaviruses. They can selectively control the epitope used and, in most cases, effectively stimulate many nAbs.

## VLPs and NPs vaccines for picornaviruses

### Vaccines against picornaviruses

Currently, picornavirus infection prevention primarily relies on inactivated and live-attenuated vaccines [1, 61, 62]. Inactivated vaccines are produced by selecting highly immunogenic and stable strains, which are then amplified, inactivated, and purified [63]. These vaccines are stable and safe but may require adjuvants to enhance immunogenicity and multiple doses for effective protection. Live-attenuated vaccines come from artificially attenuated strains obtained either through serial passage in cell cultures or by inducing mutations [64]. These vaccines typically induce strong and long-lasting immune responses through fewer doses. However, they can risk reverting to a virulent form and require careful handling and storage. For example, the primary means of FMD prevention is achieved through using inactivated vaccines, which are safe and effective, playing a crucial role in FMD control. However, inactivated vaccine production requires high-standard biosafety facilities and strict protocols to ensure complete inactivation of the virus, as incomplete inactivation comes with significant risks. Additionally, repeated vaccinations can induce antibodies against non-structural proteins. This can, in turn, make it difficult to differentiate infected from vaccinated animals (DIVA) [65]. There are also limitations involving incomplete protection and the need for cold-chain transportation. Both of these vaccines cannot quickly respond to outbreaks of new quasispecies. Picornaviruses have a high mutation rate because their replication mechanisms are prone to errors [66, 67]. This diversity within a viral population can lead to rapid adaptation and evolution, making it a significant challenge when designing vaccines. Many studies are underway to develop a vaccine that addresses these limitations.

The ideal vaccine should be safe, capable of inducing a protective immune response with a single dose, provide rapid and long-lasting immunity, be cost-effective to produce, and facilitate DIVA. VLPs and NPs vaccines are receiving widespread attention among the next generation of vaccines due to their many advantages [22, 68, 69]. VLPs and NPs vaccines do not contain viral genomes unlike inactivated and live attenuated vaccines. The absence of viral replication or disease ensures greater safety. Their production process does not require highly pathogenic viruses, which reduces biosafety risks. These two vaccines only contain the virus’s immunogenic structural proteins or epitopes, preventing vaccinated animals from producing antibodies against non-structural viral proteins. This means we can effectively differentiate between infected and vaccinated animals. The vaccines can be produced in large quantities within cell culture systems with relatively simple and controllable processes. Surface modifications on the particles can stimulate immune responses against different pathogens. The vaccine components and structures can be customised through genetic engineering or chemical changes to enhance specificity and efficacy. Antigen components can be quickly adjusted to respond to emerging strains, avoiding vaccine failure due to antigenic drift and shift. Overall, VLPs and NPs vaccines have significant advantages in terms of safety, DIVA, immunogenicity, production flexibility, and designability. This gives them a promising position within modern vaccine development. Additionally, using adjuvants can enhance the immunogenicity of vaccines and prolong the duration of effective protection [70, 71]. Selecting appropriate adjuvants may help antigens to be actively presented to dendritic cells or other antigen-presenting cells capable of cross-presentation, thereby forming an effective stimulus. The following text will review VLPs and NPs vaccines against picornaviruses and the adjuvants used.

### VLPs

#### PV

**Figure 2:**
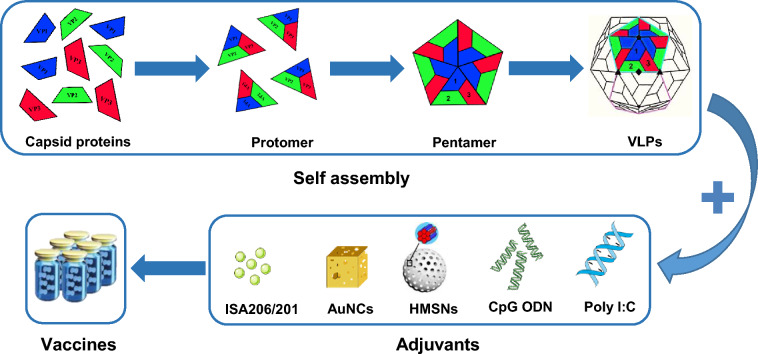
**Schematic diagram of the preparation process of VLPs vaccine.** The structure proteins (VP1, VP2 and VP3) are purified first. Due to their self-assembly characteristics, these structural proteins are assembled into protomers, followed by 5 Protomers forming pentamers, and finally forming a capsid, the VLPs. For the vaccine usage, the VLPs would then be mixed with adjuvants (e.g. ISA206/201, AuNCs, HMSNs, CpG ODN and poly I: C) [97, 107, 108, 155–157, 163, 183].

VLPs are a class of molecules that can be generated through individual expression or co-expression of viral structural proteins. They then self-assemble into spherical or tubular structures similar to viruses. Because VLPs do not contain viral genetic material, they are not contagious. These structures typically contain high-density repetitive displays of viral surface proteins, which can stimulate strong T- and B-cell immune responses. Due to the inability of VLPs to replicate, they are safer vaccine alternatives [21, 72] (Figure 2). Research on VLPs vaccines dates back to the early 1980s when researchers isolated an intermediate in the process of virus assembly during the virus purification process. This intermediate, also known as a “viral precursor” or “empty capsid”, comprises viral capsid proteins and does not contain the viral genome. With the gradual deepening of our understanding of VLPs, it has been found that this empty capsid can induce immune responses that are equal to those induced by inactivated vaccines in animals. In 1991, Montross et al. discovered that the VP1 protein of Polyomavirus, which typically assembles in the nucleus of infected mouse cells, could also assemble in the cytoplasm when treated with ionomycin [73]. Later, various viruses, such as Human papillomaviruses (HPV), HBV, HEV, influenza virus H7N9, DENV, and SARS-CoV-2, were found to have viral proteins that can self-assemble into VLPs, including capsid proteins, pre-membrane proteins, and envelope proteins [74–78]. Among them, the VLPs vaccines for HPV, HBV and HEV are currently used in the clinic, while the VLPs vaccine for SARS-CoV-2 has also been under clinical trial.

In picornaviruses, various VLPs vaccines have been developed against many viruses, including FMDV, EV71, EMCV, HAV, and PV. VLPs of PV were first produced in 1989 [84]. Urakawa et al. constructed a plasmid containing the complete 6.6 kb coding region of PV based on a baculovirus expression vector. The virus particles were recovered from extracts of the infected insect cells with no detectable RNA and no infectivity in tissue culture. After the VLPs were used to immunise the mice, structural protein antibodies including nAbs were obtained. This research showed that in picornavirus, empty particles can be made in insect cells using recombinant baculoviruses and can successfully stimulate the production of nAbs. In 1991, Ansardi et al. isolated two recombinant vaccinia viruses that express the PV P1 capsid precursor polyprotein and proteinase 3CD and infected them with HeLa cells to yield the three individual capsid proteins VP0, VP3, and VP1 [83]. However, the immune effect of these particles has not been tested. In 1997, researchers in Belgium cloned P1 and 3CD genes in a *Saccharomyces cerevisiae* inducible expression system and obtained purified empty particles to immunise the mice [85]. Serum analysis via a plaque assay showed that the empty particles elicited antibody titers comparable to those of the virions. This further confirmed the potential of VLPs to elicit immune responses, especially nAbs, at the same level as live viruses. In 2017 and 2020, *Nicotiana benthamiana* and *Pichia pastoris* expression systems were also used to express the VLPs of PV based on the P1 and 3CD genes [86, 87]. The results of the immunogenicity experiment of plant-expressed VLPs showed that the levels of nAbs induced were similar to those induced by inactivated vaccines, and the challenged animals were protected.

Recently, with further technological advancements, Fox et al. reported genetic manipulation of the virus to generate stable empty capsids for all three serotypes of PV containing several mutations in the P1 region [88]. These PV VLPs exhibited extreme stability and improved D-antigenic thermostability, maintaining 50% D-antigenicity at temperatures exceeding 60 °C. This highly stable D-antigenic conformation enables these VLPs to elicit nAb titers equivalent to those of the current inactivated polio vaccine [89]. In 2023, Sherry et al. creatively replaced the cytotoxic 3CD protein from the plasmid with the 2A peptide from porcine teschovirus (PTV), another member of *Picornaviridae* [90]. The results revealed a classic picornaviral icosahedral structure under transmission electron microscopy (TEM) and showed a VP3 antigenicity and good thermostability. With the development of VLPs vaccines against PV, VLPs can now be produced using protease-independent methods, circumventing the potential problems of cytotoxicity associated with 3CD. A widely accepted consensus now suggests that extending vaccination beyond eradication will be necessary in the foreseeable future. This will prevent the potentially disastrous reintroduction of PV into a population lacking immunity. Hence, developing new vaccines that do not rely on live virus cultivation for production is crucial. The development of new vaccines includes stable VLPs production systems using constructs that do not necessitate protease coexpression.

#### FMDV

The first FMDV VLPs appeared in 1990 as a cDNA cassette containing sequences encoding the capsid precursor P1-2A [91]. Later in 1995, Abrams et al. constructed a cDNA cassette containing the P1-2A and 3C protease, which was subsequently introduced into vaccinia virus transfer vectors [92]. After purification through sucrose gradient centrifugation, empty capsids with diameters of approximately 30 nm were observed under electron microscopy. These VLPs elicited similar antibody titres for conformation epitopes compared to whole virions and naturally empty capsids. Since then, P1-2A and 3C have become the predominant choices when it comes to researching FMDV VLPs [79, 93–96]. To further address the issue of low water solubility in recombinant virus capsid proteins, Lee et al. developed a SUMO fusion protein system to determine whether SUMO modification was the key to improving the water solubility of viral proteins [97]. Then Guo et al. used an improved SUMO fusion protein system to express FMDV VLPs in *Escherichia coli* (*E. coli*) and tested their immune response in guinea pigs, swine, and cattle [29]. The results showed that the FMDV-specific antibody response, nAbs response, T-cell proliferation response, and cytokine interferon-γ (IFN-γ) secretion were stimulated. The complete protection of these animals from homologous FMDV challenge was reported, indicating the future of FMDV VLPs as vaccines. Additionally, various expression systems, such as B/IC: *Sf*-21, *Samia cynthia ricini*, *Nicotiana benthamiana*, *Agrobacterium tumefaciens*, HEK-293A, HEK-2936E, and HEK-293 T, have all been used to express FMDV VLPs based on the gene sequences of P1-2A or P1-2A with 3A [98–104]. In 2023, Tan et al. reported the first method for inducing chromosome expression of the FMDV capsid protein in BHK-21 cells, simplifying the production of FMDV VLPs vaccines in mammalian cells [105].

Currently, greater attention is being given to further enhancing the thermostability of VLP vaccines, and biomineralisation technology has also been applied in this research [106, 107]. For example, Guo et al. encapsulated FMDV VLPs using calcium phosphate, forming particles with approximate diameters of 80 nm [107]. After heat treatment at 37 °C for 4 days, the biomineralised VLPs remained immunogenic and produced particular antibodies and nAbs with a high protection rate. This year, a zeolitic imidazole framework was used to encapsulate FMDV VLPs, resulting in particles with approximate diameters of 75 nm. [108]. Furthermore, these particles can elicit a stronger immune response than VLPs alone, even when exposed to 37 °C for 7 days. This evidence further supports the potential application of biomineralisation technology in developing VLP-based vaccine products, aiming to reduce the high costs associated with cold chain transportation.

#### EV

In a 2008 study on enterovirus, Chung et al. coexpressed the P1 and 3CD proteins of EV71 using the B/IC system, which facilitated their assembly into VLPs resembling natural EV71 structures. The immunogenic response elicited by these VLPs was found to be superior to that elicited by denatured VLPs and heat-inactivated viruses [80]. The VLPs also had a better protective effect on newborn mice and produced greater nAbs titers. This result suggested that maintaining the structural integrity of VLPs is crucial for an effective immune response. In 2010, Chung et al. modified the promoter into a weaker CMV and IE-1 promoter and carried out the transformation [109]. Additionally, different cell lines were selected, and the expression conditions were optimised, which significantly improved the expression of EV71 VLPs (from 1.5 to 64.3 mg/L) and the structural stability. This is very important for the immunity of VLPs and lays a foundation for further development of EV71 VLPs vaccines. Later, in 2020, Yang et al. constructed and expressed the codon-optimised P1 and 3C genes of EV71 in *Pichia pastoris* to produce EV71 VLPs with a high yield and simple manufacturing process by achieving an expression level of 270 mg/L [110]. Wang et al. then comprehensively evaluated the nonclinical immunogenicity, efficacy, and toxicity of these high-yield EV71 VLPs in rodents and nonhuman primates [111]. The immunogenicity assessment showed that the EV71 VLPs vaccine elicited high and persistent nAbs responses comparable to those of a licensed inactivated vaccine. The immune sera of vaccinated mice also exhibited cross-neutralisation activities to the heterologous subtypes of EV71. In addition, expression systems, including *Saccharomyces cerevisiae*, HEK-293A, and *Flammulina velutipes* have also been employed to express the VLPs of EV71 based on the gene sequences of P1 and 3CD or 3C [112–114]. The data demonstrated the high immunogenicity of EV71 VLPs and strongly supported the further application of VLP-based EV71 vaccines in humans.

#### SVA

In 2020, Mu et al. developed VLPs for SVA using a prokaryotic expression system and evaluated their immunogenic potential [115]. Following characterisation by affinity chromatography, sucrose density gradient centrifugation, ZetaSizer, and TEM, the VLPs emulsified with the ISA 201 adjuvant were administered intramuscularly to the pigs. The inactivated SVA vaccine was also injected in the same way as the control group. The results showed that the levels of neutralising and specific antibodies and IFN-γ in the VLPs group were similar to those in the inactivated SVA group. Additionally, after the challenge, the protection rate of the VLPs group was similar to that of the inactivated SVA group. In 2021, Wu et al. developed SVA VLPs through a prokaryotic expression system based on the genomic sequence of the field isolate CH-FJ-2017 [116]. TEM revealed that the three structural proteins of SVA (VP0, VP1 and VP3) could self-assemble into VLPs with a diameter of approximately 25–30 nm in vitro, and the animal experiment results showed that the VLPs could effectively stimulate guinea pigs to produce a high level of antigen-specific nAbs. In 2024, Zhang et al. first used a baculovirus expression vector system to develop SVA VLPs [117]. In conclusion, these reports confirmed that SVA VLPs could elicit high levels of innate and acquired immune responses and have great potential as vaccine candidates for the defence and elimination of SVA.

#### EMCV

In 2010, Jeoung et al. first developed EMCV VLPs by expressing a plasmid containing the P12A and 3C gene sequences from the EMCV K3 strain in 293FT cells. They then evaluated the immune response elicited by these VLPs in vivo [118]. The obtained VLPs were identified as particles of approximately 30–40 nm under TEM. The results of animal experiments showed that the levels of cytokines, including the Th1 indicators (IL-2, TNF-alpha, and GM-CSF) and the Th2 indicators (IL-4 and IL-10), had increased. High levels of nAbs were induced, and a high protection ratio (90%) after challenge with EMCV-K3 was observed. This study indicated that the VLPs of EMCV successfully activated cellular and humoral immune responses in animals and produced nAbs with sound protective effects. In 2011, this laboratory published another work on developing EMCV VLPs in *Spodoptera frugiperda* (*Sf*-9) cells, showing a 90% protection rate in mice. In pigs, increased nAbs at a high level were observed following two immunisations. In 2023, Zhang et al. constructed and assembled EMCV VLPs using an *E. coli* system and verified their efficient protective effect against viruses. Thus, with these results, we might expect VLPs with good immunogenicity to be novel vaccines against EMCV and effective at controlling EMCV infection on pig farms.

#### Other picornavirus VLPs

There are also some studies on the VLPs of other picornaviruses, such as duck hepatitis A virus (DHAV), CVA16, and CVB3, but the number of studies on their VLPs is limited. Thus, there is a lack of systematic information. Here, we provide a short description and brief summary.

In 2018, Wang et al. developed VLPs for DHAV by expressing the structural polyprotein precursor gene P1 and the protease gene 3CD using a baculovirus expression system [119]. The animal experiment showed that when the ducklings were vaccinated with VLPs, the commercial attenuated vaccine A66 and the two control groups, this induced similarly high humoral immune responses in both the VLPs and A66 groups, which were much greater than those in the vector and PBS groups. Additionally, the protective effects in the VLPs group were comparable to those in the A66 group, as all the ducklings in both groups survived without exhibiting any clinical signs. This study provides reference data on the immunogenicity of DHAV VLPs, demonstrating that VLPs, as a new-generation vaccine, exhibit significant potential for application in the development of picornavirus vaccines.

In 2012, Liu et al. developed CVA16 VLPs by coexpressing the P1 and 3CD proteins of CVA16 in insect cells utilising recombinant baculoviruses [120]. These VLPs of CVA16 were spherical particles approximately 30 nm in diameter and contained processed VP0, VP1, and VP3. According to the results of the serological tests, high levels of specific antibodies and nAbs were detected in VLPs-immunised mice. After challenging the mice with the homologous strain CVA16/SZ05, all the mice in the VLPs group survived and remained healthy. Furthermore, they also used the heterologous strain CVA16/GX08, which is more virulent than CVA16/SZ05, to challenge neonatal mice. Anti-sera treatment was given to the mice at 24 h post-infection. The survival rate for the anti-VLPs sera recipient group was 88.9%. This research effectively demonstrated the preventive and therapeutic effects of VLPs.

In a 2012 study on CVB3, Zhang et al. engineered two distinct recombinant baculoviruses: one containing the complete coding region of CVB3 and the other comprising the four individual coding regions for the viral structural proteins VP1-4. They expressed capsid proteins in insect cells infected with recombinant baculoviruses and subsequently confirmed the presence of VLPs in sucrose fractions using electron microscopy [121]. After vaccinating SWR (H-2(q)) mice with VLPs or an attenuated CVB3 vaccine, antibodies comparable to those of CVB3 capsid proteins were induced after the first boost. A greater immune response and better protection were observed in the VLPs group with the complete CVB3 coding region than in the VLPs group with only structural proteins.

Due to the immune responses elicited by VLPs of various picornaviruses, the results of these serological tests and animal challenge protection tests have confirmed VLPs' great immunogenicity and ability to serve as preventive vaccines for viral diseases in humans and animals.

However, as a vaccine candidate, VLPs production yields play a pivotal role in vaccine development, as higher yields are often associated with increased vaccine availability and reduced production costs. There's still a challenge to achieve high VLP yields for picornaviruses due to the intricate nature of VLP assembly and the limitations of the expression systems used. The cold chain is also still essential for storing and transporting certain kinds of VLPs. Thermal instability can lead to VLP degradation and loss of immunogenicity, ultimately impacting vaccine efficacy. It is essential to ensure thermal stability for FMDV vaccines commonly used in areas with limited cold chain infrastructure. One such example is the work by Porta et al. [122], which exemplifies attempts to design recombinant picornavirus capsids as vaccines rationally. This study explores strategies to enhance VLPs yields and improve thermal stability by modifying capsid proteins and optimising expression systems. By employing rational design principles such as protein engineering and structure-based approaches, researchers aim to overcome the limitations associated with VLPs production and enhance the immunogenicity of VLP-based vaccines. Similarly, Lin et al. also developed a production and chromatographic purification process to increase the yield of VLPs in EV71. This technology has a better or equivalent recovery rate and purity than purifying live EV71 virus particles [123]. In addition, biomineralization technology has been applied in the study of VLPs in FMDV to improve the thermal stability of particles [106–108]. Incorporating these findings is crucial for enhancing our understanding of VLP-based vaccine development and guiding future research efforts to overcome the challenges related to VLPs production, especially for picornavirus vaccines.

### NPs

#### Peptide-based NPs

NPs are particles that can self-assemble from proteins into special shapes (such as polyhedra, flower-like, porous) through intermolecular electrostatic forces, electric dipole interactions, external electric field interactions, and van der Waals forces [124, 125]. In a broader sense, NPs are typically particles ranging in size from 1 to 100 nm, which allows them to concentrate more easily in lymph nodes, thereby enhancing immune responses [54, 126, 127]. In this article, we adopt a narrow definition of NPs by saying that their composition primarily consists of oligomeric non-viral proteins that can self-assemble in nature. At the same time, VLPs utilise viral proteins [128]. The shape and composition of NPs can vary based on their intended use. Compared to VLPs, NPs offer several advantages: good biocompatibility, uniformity, and the ability to bind different antigens [54]. As a potential alternative to VLPs, the highly oligomeric non-viral proteins that constitute NPs are often enzymes or dynamic equilibrium proteins, making them easier to produce. Their uses include acting as adjuvants when mixed with antigens, encapsulating antigens, and serving as antigen display platforms. Here, we primarily focus on NPs as vaccines for antigen display platforms. For NPs against picornaviruses, the fundamental theory is that the epitopes of the virus can trigger an immune response in the host, thereby providing immune protection against potential viral infections. In this case, we believe the multimerisation of viral epitopes should also be considered a part of NPs vaccines against picornaviruses. However, the framework of NPs is similar to a platform for displaying these epitopes, which are simple peptides from the structural and nonstructural proteins of picornaviruses. Various backbones can be used to develop NPs, and we would like to focus more on the different frames in this section rather than different viruses, after we systematically discuss epitopes in the multimerisation section.

**Figure 3:**
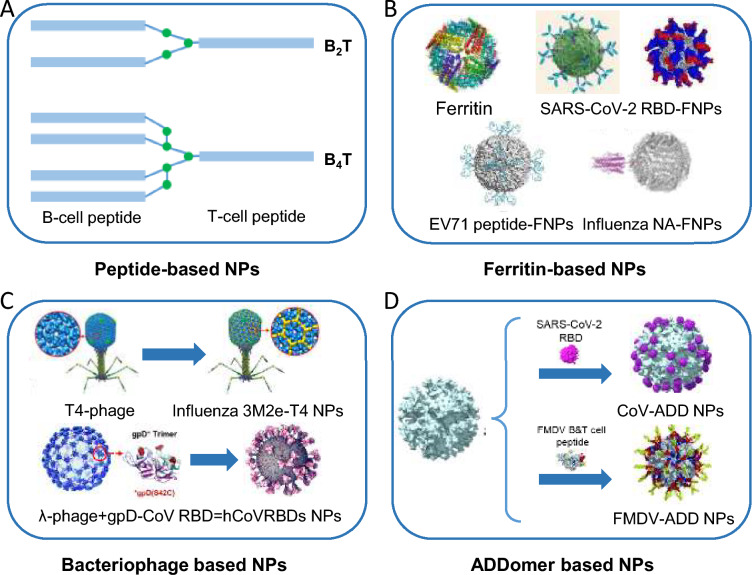
**Schematic diagram of NPs**. **A** Peptide-based NPs [130, 133, 134, 194]; **B** Ferritin based NPs [139, 144–150]; **C** Bacteriophage based NPs [155–160]; (D) ADDomer based NPs [163–165].

Multimerisation, especially dendrimers, is a natural way to mimic antigen presentation and has been successfully used in vaccine preparation [129]. This dendrimer peptide repeats the branching reaction of amino acids based on the core motif to obtain dendrimer polypeptide macromolecules. This strategy has also proved successful in vaccine development for the picornavirus FMDV (Figure 3).

In 2008, Cubillos et al. designed and synthesised a dendritic peptide, B4T, which comprises four B-cell epitopes from the G-H loop (amino acid residues 136–154 in VP1) and one T-cell epitope from the 3A region (amino acid residues 21–35). This design was based on the sequence of the FMDV serotype C isolate C-S8c1 [130]. This dendrimer particle demonstrated a robust protective effect, as evidenced by the four immunised pigs (intramuscular injection with 1.4 mg of dendrimer peptide twice, emulsified with complete Freund’s adjuvant at day 0 and with incomplete Freund’s adjuvant at day 21) that did not develop significant clinical signs in response to FMDV challenge (at day 18 post boost) and exhibited no signs of viral transmission to other pigs. High titers of FMDV nAbs and activated FMDV-specific T-cells were observed. These results indicated that with the dendritic arrangement of B-cell epitopes and T-cell epitopes, the particles stimulated both cellular and humoral immunity and provided solid protection against viral challenge to pigs with a dose of 1.4 mg per pig with adjuvants. As analysed in the study by Jiao and Wu, peptide vaccines exhibit a dose-dependent protective effect against FMDV infection, particularly showing lower protection rates compared to inactivated vaccines when the injected dose is less than or equal to 1 mg [131]. Additionally, it has also been reported that peptide vaccines in dimeric or multimeric forms provide more robust protection than single-epitope vaccines [132]. Cubillos et al.'s research used a 1.4 mg dose of B4T, the multimer for injection. They emulsified it with adjuvants, which helped to provide effective protection against FMDV in animals. Later, in 2016, Blanco et al. from the same consortium further improved the immune response to dendric particles by downregulating the particle from four B-cell epitopes to two [133]. Groups of B2Ts immunised pigs showed protection rates greater than those of B4T-immunised pigs. Viral shedding was prevented entirely in the B2T (thi) group. In addition, bivalent groups showed significantly greater T-cell responses (IFN-γ) than the tetravalent group did (*p* < 0.05). In 2017, this consortium further proved this dendric strategy in cattle [134]. After three vaccine doses, full protection with no clinical signs was observed in the B4T-immunised group. In addition, the long-term protective effect of B2T (mal) was verified to be elicited by the dendrimer in 2020 [135]. After one or two doses of B2T (mal), the immunisation conferred a long-lasting reduced susceptibility to FMDV infection up to 136 days (19/20 weeks) post-boost.

These findings have explored the possibility of vaccine design strategies based on B- and T-cell epitope combinations and the potential of multimerisation design of virus peptide-based vaccines. The short- and long-term immune responses elicited and protection against challenge proved this strategy’s immunogenicity. Since this method is usually used in biomedical products or as a drug delivery platform [136, 137], more research is needed for further study of multimerisation peptide-based vaccine design.

#### Ferritin-based NPs

Ferritin, a ubiquitous, multisubunit iron storage protein first found in the horse spleen, was later found in mammals, plants and microorganisms [138–142]. Ferritin is mainly composed of a mineral core and a protein shell. This protein shell is self-assembled from 24 polypeptide chains, of which three chains form a trimer, and eight trimers form a roughly spherical hollow nanocage with an external diameter of 12 nm and an inner diameter of 8 nm [139]. Ferritin exhibits remarkable stability, withstanding high temperatures and various denaturing agents, and is sensitive to pH. The exterior surface and subunit interface of ferritin can be modified without affecting its assembly. Any ligand with targeting markers can be added to ferritin chemically or via recombinant genetic techniques. Therefore, ferritin has become an ideal multifunctional nanocarrier and an ideal platform for antigen presentation and vaccine development [143]. For example, Tai et al. designed a self-assembling ferritin-based NPs vaccine against the SARS-CoV-2 Omicron variant. This NPs vaccine with the receptor binding domain (RBD) (residues 331 aa-524 aa) of the Omicron spike protein provided a robust humoral immune response and effective protection against this variant. This also demonstrated the potential of NPs vaccines to respond quickly to emerging and reemerging viruses in the future [144]. In addition, ferritin has also been applied in vaccine research for many other viruses, such as influenza virus, human immunodeficiency virus (HIV), HPV, Hepatitis B virus (HBV), and Zika virus [145–149] (Figure 3).

Among the viruses of the *Picornaviridae* family, ferritin has been the subject of many related vaccine studies. For FMDV, in 2020, Chen et al. used a baculovirus expression system to express NPs and evaluated their immune responses in mice [34]. Four recombinant proteins were successfully expressed, namely, VP1, VP1-ferritin (VP1-Ft), G-H loop-ferritin (G-H loop-Ft), and ferritin. Between the VP1 and VP1-Ft groups, the VP1-Ft group (66.7%) had a greater protection rate than the VP1 group (55.6%). Furthermore, the G-H loop, a widely used immunogenic peptide located in VP1, is a hypervariable region with the most antigenic site that triggers nAbs. The G-H loop-Ft group showed a protection rate of 77.8% against FMDV in mice, greater than that of the VP1-Ft group. Additionally, the G-H loop-Ft group had greater nAbs titres, IFN-γ levels and no tissue lesions versus the VP1 and VP1-Ft groups. This finding suggests that for FMDV vaccines, the G-H loop region has a superior capacity to elicit cellular and humoral immune responses compared to that of VP1. This finding indicates that the G-H loop may be a more favourable candidate for future NPs studies than VP1. Ferritin NPs have good immunogenicity and could be potential vaccine candidates.

Moreover, the ferritin-based NP vaccine strategy has been employed against EV71, incorporating epitopes fused with ferritin, as illustrated in Figure 3. In 2019, Wang et al. designed and expressed nine different ferritin-EV71 NPs in a prokaryotic system and evaluated their immune responses in mice [150]. The researchers chose the EV71 VP1 linear epitope (residues 208 aa-222 aa in VP1) and two truncated epitopes with different lengths (residues 215 aa-218 aa and 213 aa-220 aa in VP1) to fuse with three different regions of ferritin (the N- and the C-termini and the loop zone). As expected, these nine NPs were stable after heating at 70 °C for 5 min and showed no morphological changes at different pH values (pH = 5.0, 6.0, 7.0 and 8.0), proving ferritin's good stability. The findings revealed that the loop zone group exhibited the highest neutralizing antibody (nAb) titres and protection rate (100%). Furthermore, all groups with epitopes displayed on the surface of ferritin demonstrated higher antibody titres than those displayed within the particles. Researchers have explained the differences observed between the N-terminal and loop zone groups. The different structures of these two sites might lead to differences in epitope density and antigen presentation, which further affects the immunogenicity of the particles. Among the epitopes of different lengths, the longest epitope had the highest antibody titres. In contrast, the loop zone group's longest and second longest epitopes both showed 100% protection against virus challenge. This experiment further clarified the influence of the length of the FMDV epitope on its immunogenicity and the best region of ferritin to display the epitopes.

These studies systematically explored the optimised site of ferritin as an epitope display platform in vaccine research. They demonstrated the importance of the length and fusion site of selected peptides, which could be crucial to the immunogenicity of NPs.

#### Bacteriophage-based NPs

Bacteriophages, viruses that infect bacteria, fungi, actinomycetes, and other microorganisms, are present in most prokaryotes. They are mainly composed of proteins and nucleic acids. In 1985, Smith GP first inserted a foreign gene into gene III of a filamentous phage so that the polypeptide encoded by the target gene could be displayed on the phage surface as a fusion protein, thus creating a bacteriophage display technology [151]. To date, this display technology has been successfully used to display peptides of viruses on various phages, such as M13 phage, λ phage, T4 phage, and T7 phage [152–160] (Figure 3). From a structural perspective, the empty particles of the bacteriophages can be referred to as VLPs. However, these particles serve as a platform for presenting heterologous antigenic epitopes. Therefore, following the description of Tao et al., we classify them as NPs [161]. Among these phages, T4 and T7 have already been employed in picornavirus research. The remarkable feature of the T4 bacteriophage is that it can fuse two foreign polypeptides/proteins with entirely different properties from SOC and HOC sites on the capsid protein simultaneously. The particles are assembled within host cells, displaying a wide range of polypeptides/proteins. T7 bacteriophage display technology was established in the late 1990s. The 10B protein on its capsid is often used to construct phage display systems. The production of the T7 bacteriophage is speedy, saving considerable time and facilitating research.

In 2008, Ren et al. used a T4 bacteriophage NPs surface gene-protein display system (T4-S-GPDS) to create T4-P1 and T4-3C particles displaying the P1 and 3C sequences of the FMDV serotype O strain GD-10 respectively [155]. These particles were administered to various groups of mice orally or via subcutaneous injection, with some groups receiving an additional adjuvant. After challenge with a lethal dose of virulent FMDV derived from cattle, all mice achieved 100% protection following immunisation, regardless of whether it was administered orally or through injection. In addition, two pigs immunised with a mixture of phage T4-P1 and phage T4-3C were protected despite living with infected pigs. Based on a meta-analysis article, the protective effects on pigs were statistically non-significant from those of mice [MH = 0.56, 95% CI (0.20, 1.53), *P* = 0.26]; thus, the mice could replace pigs as FMDV vaccine models to test the protective effect of the vaccine [162]. This explains why Ren et al. used a mice model to test the protection efficiency of the T4-P1 and T4-3C particles and used pigs only to verify the effect of the NPs on virus transmission. Later, in 2017, Xu et al. used T7 bacteriophage-based NPs displaying the G-H loop peptide of the FMDV capsid protein VP1 (T7-GH) by inserting the G-H loop coding sequence into the T7 Select 415-1b vector [160]. The T7-GH particles, in combination with the adjuvant Montanide ISA206, were administered via injection to pigs. However, upon challenge with a virulent homologous virus, only four of the five pigs immunised with T7-GH demonstrated protection. In 2021, Wu et al. engineered the T7 phage capsid to express the VP1 of the FMDV AKT-III strain and evaluated its potential as an FMD vaccine [159]. Following immunisation, the mice quickly generated high levels of FMDV antibodies, which persisted for an extended period. A high level of IFN-γ was also observed, indicating a robust cellular immune response alongside the humoral immune response. In a subsequent study in 2022, Wong et al. used the bacteriophage T7 to display the VP1 epitope (residues 131–170) of FMDV [157].

The above studies suggested that displaying the epitopes or capsid proteins of picornaviruses on the surface of bacteriophage particles could be a promising strategy for eliciting protective cellular and humoral immune responses against virus challenge. This leads to the optimistic prediction that these recombinant bacteriophage NPs may become a safe and productive vaccine against picornavirus.

#### ADDomer-based NPs

The ADDomer platform, a novel approach to vaccine development, was first introduced into literature in 2019 [163]. In collaboration with Oracle, a research team from the French National Centre for Scientific Research (CNRS) developed the self-assembled vaccine synthesis element ADDomer. ADDomer is an NPs scaffold derived from an adenovirus polyprotein capable of incorporating and presenting immunogenic epitopes of pathogens. This vaccine technology platform demonstrates impressive thermal stability, overcoming, to some extent, a longstanding challenge in vaccine development. Additionally, this approach dramatically simplifies vaccine design and production processes. It offers promising opportunities for preventing and controlling infectious diseases. In 2022, Chevillard et al. used ADDomer to display the glycosylated RBD of SARS-CoV-2 linked by SpyTag/SpyCatcher [164] (Figure 3). This decorated dodecahedron was injected into mice and induced a significant specific humoral response.

In the case of picornavirus, in 2022, ADDomer was also successfully used against FMDV [165] (Figure 3). Two dominant B-cell epitopes (residues 129 aa-160 aa and 200 aa-213 aa, VP1) and a dominant T-cell epitope (residues 16 aa-44 aa, VP1) of type O FMDV were inserted into the ADDomer variable loop (VL) and arginine-glycine-aspartic acid (RGD) loop. Following expression using the baculovirus system and confirmation via Western blot, mass spectrometry, and TEM, the immunogenicity of this recombinant particle was evaluated using a murine model. At 28 dpi, the percentage of CD4 + T-cells in the spleen of mice in the experimental groups was greater than that in the PBS group, as detected by flow cytometry. Additionally, mice in the experimental group showed a greater level of IL-4 than those in the inactivated vaccine group and a significantly greater level of IFN-γ secretion than those in the PBS group. The ADDomer and FMDV epitopes combined stimulated more IFN-γ secretion than ADDomer alone. FMDV-specific antibodies were detected in the immunised mice. These results suggest that the NPs effectively induced both cellular and humoral immune responses and that the B-cell and T-cell epitopes of these particles were successfully delivered using this platform. These studies demonstrated the advantages of ADDomer as an antigen display platform and its potential in vaccine development. Thus, this plug-and-play vaccine platform looks set to be a promising new tool for combating emerging pathogens.

## Adjuvants

### Mineral adjuvants

Adjuvants, also known as immune modulators or enhancers, can nonspecifically enhance the body’s specific immune response to antigens and play an auxiliary role. They are vaccine additives that can induce long-term and efficient specific immune responses, improve the body's protective ability, and reduce the amount of vaccine used. They, in turn, reduce the production cost of vaccines (Figure 2). Over the years, various materials have been investigated as potential adjuvants to enhance the immunogenicity and stability of VLPs and NP vaccines. Here, we will discuss adjuvants used in the VLPs and NPs against picornaviruses using three categories: mineral adjuvants, oil adjuvants, and microbial derivative adjuvants.

Among these materials, mineral adjuvants such as gold and silicon have received significant attention. Gold is consistently regarded as one of the most promising nanomaterials for cutting-edge vaccine research. This is due to its dependable surface functionalisation, biocompatibility, customisable size and shape, and exceptional optical properties [166]. The Au-NPs are first synthesised by a reduction reaction using Turkevich or Brust-Schrifffins procedures followed by surface modification and stabilisation [167]. In addition, Au-NPs have also been used in the vaccine research of many other viruses, such as SARS-CoV-2, HIV, hepatitis E virus (HEV), and dengue virus (DENV) [30, 168–170]. In 2021, in picornavirus, Teng et al. mixed Au nanocages (Au-NCs) as adjuvants with VLPs at a ratio of 1:2.5 (w/w) to form the VLP-AuNCs with a diameter of approximately 90 nm [171]. VLP-AuNCs promoted the secretion of IL-6, IL-1β, and TNF-α, triggering a strong immune response against FMDV in mice and guinea pigs. The protection rate against FMDV in guinea pigs in the VLP-AuNCs group was greater than in the VLPs group. In addition, the ICP-MS results showed that these particles mainly accumulated in the spleen and liver of immunised mice, with no significant histopathological changes.

Silicon dioxide is one of the most common forms of silicon in nature. Its manufacturing method is simple, low cost, and shows good biocompatibility [172]. Among the various forms of silicon dioxide, hollow mesoporous silica nanoparticles (HMSNs), which belong to mesoporous silica nanoparticles (MSNs), have advantages such as adjustable particle and pore sizes, relatively large surface areas, and straightforward functionalised surfaces containing a large amount of silicon hydroxide [173]. Due to these advantages, HMSNs have also received much attention in the development of viral vaccines against porcine circovirus type 2 (PCV2) and bovine viral diarrhoea virus (BVDV) [174, 175]. In 2019, Bai et al. mixed the HMSNs as a nanoadjuvant with FMDV VLPs to make the HMSNs/VLPs complexes approximately 1 µm [176]. After the immunisation in guinea pigs, the HMSNs/VLPs groups demonstrated a significantly greater FMDV-specific antibody response in the 14th week than the VLPs/Freund’s complete adjuvant group, indicating the superior slow-release effect of HMSNs. Moreover, the protection rate against FMDV in guinea pigs in the HMSNs/VLPs group reached 80%. This research suggested that HMSNs facilitate antigen presentation and can decelerate antigen release, thereby sustaining a prolonged immune response.

### Oil adjuvants

Despite advances in adjuvant development, oil adjuvants remain the most widely employed in animal vaccine preparation to enhance immune responses. For instance, Montanide™ ISA206 VG (ISA206) from the French company Seppic has been used in FMD vaccines for over two decades. It elicits robust short-term and long-term immune responses, primarily mediated by nAbs, while exhibiting low viscosity and minimal side effects. Recently, Seppic developed Montanide™ ISA201 VG (ISA201) based on ISA206, which has been shown to induce superior humoral and cellular immune responses against FMD compared to ISA206, ultimately enhancing protection against viral challenge.

### Microbial derivative adjuvants

In addition, microbial derivative adjuvants immunostimulating molecules such as polyriboinosinic:polyribocytidylic acid (poly I:C) and CpG oligo deoxy nucleotides (CpG ODNs), have also been commonly used. After the pairing of synthetic polyriboinosinic and polyribocytidylic acid, poly I:C is a highly effective interferon inducer that is a double-strand product [177]. CpG-ODNs refers to an oligodeoxynucleotide containing a CpG motif, which is a specific nucleotide sequence structure composed of unmethylated cytosine and guanine [178]. In 2015, Terhuja et al. used CpG or poly I:C as adjuvants to recombinant FMD VLPs encoded by FMDV type O/IND/R2/75 polyprotein genes expressed in Sf9 cells and compared their ability to induce a protective immune response in guinea pigs [179]. The results demonstrated that the VLP + CpG group exhibited elevated levels of IgG2 compared to those in the conventional vaccine (inactivated vaccine) group, as well as a greater protection rate against FMDV in guinea pigs than did the VLP + ISA206 and VLP + poly I:C groups. These findings highlight the advantages of utilising nucleotide adjuvants. Since VLPs consist entirely of proteins, incorporating such nucleotide adjuvants is crucial for enhancing humoral and cellular immune responses in vaccinated animals.

Overall, we are still longing for an ideal adjuvant that has few side effects for animals, a stable and lasting immune effect, low production costs, and can produce an appropriate immune response to achieve a protective effect. Thus, continuous attention is still needed to identify new adjuvants that can further improve cellular and humoral immune levels with high efficiency and low toxicity.

## Conclusion and prospects

In this review, we described a large variety of VLPs and NPs of picornavirus along with their immune effects in animals, including VLPs of PV, FMDV, and EV71 and NPs such as peptide-based NPs, ferritin NPs, bacteriophage NPs, ADDomar NPs, and adjuvants that are commonly used in VLPs and NPs vaccines against picornaviruses. VLPs vaccines for HBV, HEV and HPV have already been used worldwide [180–182], while for picornavirus, the FMDV VLPs vaccine was licensed by the Chinese government in 2021, which indicates the great potential of this VLPs vaccine. Self-assembling scaffolds have been used to present virus antigens, such as structural proteins or epitopes, for studies of picornavirus vaccination, mainly in EV71, FMDV and PV. For the NPs vaccine, in all cases, antigen immunogenicity was increased by multivalent presentation and, in some cases, was even comparable to that of the inactivated and attenuated vaccines. On the other hand, the rise of computational design to generate novel self-assembling proteins has led to the development of vaccines for acute human-infected viruses such as SARS-CoV-2, HIV, and influenza virus [183–185]. In addition, studies have emerged on identifying key viral epitopes. Based on available information, clinical trial data for VLPs and NPs of picornaviruses are minimal [186]. Aside from the abovementioned VLPs vaccine for FMDV, there is only one clinical trial for PV VLPs found, CS-2036, which was conducted by CanSino Biologics Inc., under clinical trial phase I, approved by the Therapeutic Goods Administration (TGA) of Australia [187].

**Figure 4:**
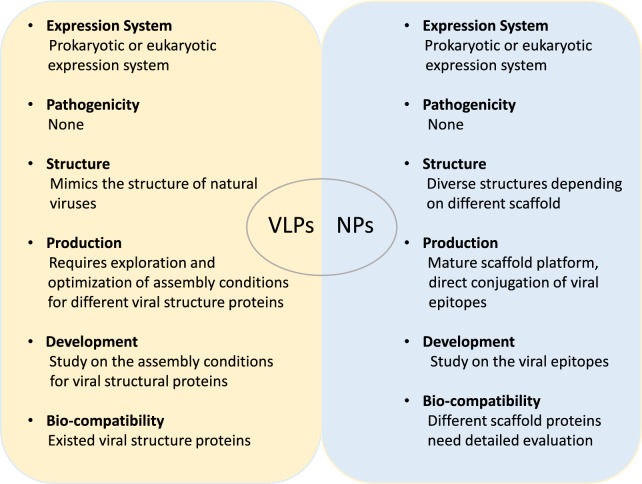
**Differences between VLPs and NPs.** The differences between these two vaccines are compared in terms of their expression system, pathogenicity, structure, production, development, and biocompatibility.

Experimental research has shown promising results for nanovaccine technology. Further efforts from the nanomaterials, immunology, virology, and pharmaceutical industries will jointly promote the development of VLPs and NPs vaccines. In the past few decades, the rapid development of nanotechnology has laid the foundation for nanovaccine development. Given the common traits of viral infectious diseases, including the unpredictable re-emergence of known viruses, the high incidence rate, and the profound socioeconomic impact of new diseases caused by unknown viruses, it is crucial to emphasise the significance of vaccine research and development. The development of nanotechnology provides a method for solving problems as soon as possible. The comparisons between VLPs and NPs can be seen in Figure 4. VLPs and NPs vaccines are very promising in the biomedical field due to their similar structure to natural viruses, good safety, immunogenicity, stability, and ability to display exogenous proteins. VLPs and NPs vaccines can stimulate the body to produce sufficient protective antibodies against viruses, making them powerful and flexible vaccine development platforms.

However, although VLPs and NPs vaccines have shown excellent prospects, some shortcomings cannot be ignored. First, the most effective design scheme for these vaccines has not yet been fully explored, and further research is needed on their targeting, biological distribution, and potential to cause unexpected cytotoxicity. 30–99% of the particles will be distributed to livers after administration [188]. While they penetrate target cells through their surface receptors, the targeting and distribution in vivo are not accurate [189]. This distribution pattern can impact their immune efficacy and safety. Additionally, their retention and accumulation in specific tissues or organs may lead to toxic or inflammatory reactions [190]. Furthermore, particles with a diameter less than 15 nm may be rapidly cleared by the immune system, resulting in a shorter residence time within the body [191]. This, in turn, affects their immunogenicity. Following exocytosis, NPs have demonstrated the capability to traverse crucial in vivo barriers, including the blood–brain barrier, leading to unforeseen cytotoxic effects. The next point to consider is how we can address the challenge of scaling up vaccine production. In developing vaccines, it is important to consider cost as a necessary factor. In addition, the research and development platform for VLPs and NPs vaccines requires professional production facilities and highly skilled labour, which is also a challenge for some resource-limited countries. Finally, there is the issue of vaccine transportation and storage. Currently, most vaccines require cold chain storage, which increases vaccine production and distribution complexity. However, as mentioned in our article, with the advancement of technology, these problems are also gradually being solved. In short, VLPs and NPs vaccines are a promising means of combating infections caused by picornaviruses and other infectious viruses.

## Data Availability

Data sharing is not applicable to this article as no datasets were generated or analysed during the current study.
